# Expression of Concern: miR-802 participates in the inflammatory process of inflammatory bowel disease by suppressing SOCS5

**DOI:** 10.1042/BSR-2019-2257_EOC

**Published:** 2023-06-05

**Keywords:** inflammatory bowel disease, mice colitis model, miR-802, SOCS5, TH17, TNF-α

The Editorial Office has been made aware of potential issues surrounding the scientific validity of this paper, hence has issued an expression of concern to notify readers whilst the Editorial Office investigates. It has been noted that the Western blots of Figure 5C appear similar to those in a retracted publication by a different author group (DOI: 10.1042/BSR20194427 Figure 2E), and that there are several repeated patterns in the data points of Figure 2C (inhibitor-NC). The authors have previously requested to correct Figure 6A, and this request was under consideration at the time these additional concerns were raised by readers.

